# Meso-Microporous Carbon Nanofibrous Aerogel Electrode Material with Fluorine-Treated Wood Biochar for High-Performance Supercapacitor

**DOI:** 10.3390/gels10010082

**Published:** 2024-01-22

**Authors:** Md Faruque Hasan, Kingsford Asare, Shobha Mantripragada, Victor Charles, Abolghasem Shahbazi, Lifeng Zhang

**Affiliations:** 1 Department of Nanoengineering, Joint School of Nanoscience and Nanoengineering, North Carolina A&T State University, Greensboro, NC 27401, USA; 2 Department of Natural Resources and Environmental Design, College of Agriculture and Environmental Sciences, North Carolina A&T State University, Greensboro, NC 27411, USA

**Keywords:** aerogel, nanofiber, electrospinning, biochar, supercapacitor, sustainability

## Abstract

A supercapacitor is an electrical energy storage system with high power output. With worldwide awareness of sustainable development, developing cost-effective, environmentally friendly, and high-performance supercapacitors is an important research direction. The use of sustainable components like wood biochar in the electrode materials for supercapacitor uses holds great promise for sustainable supercapacitor development. In this study, we demonstrated a facile and powerful approach to prepare meso-microporous carbon electrode materials for sustainable and high-performance supercapacitor development by electrospinning polyacrylonitrile (PAN) with F-treated biochar and subsequent aerogel construction followed by stabilization, carbonization, and carbon activation. The resultant carbon nanofibrous aerogel electrode material (ENFA-FBa) exhibited exceptional specific capacitance, attributing to enormously increased micropore and mesopore volumes, much more activated sites to charge storage, and significantly greater electrochemical interaction with electrolyte. This electrode material achieved a specific capacitance of 407 F/g at current density of 0.5 A/g in 1 M H_2_SO_4_ electrolyte, which outperformed the state-of-the-art specific capacitance of biochar-containing electrospun carbon nanofibrous aerogel electrode materials (<300 F/g). A symmetric two-electrode cell with ENFA-FBa as electrode material showed an energy density of 11.2 Wh/kg at 125 W/kg power density. Even after 10,000 cycles of charging-discharging at current density of 10 A/g, the device maintained a consistent coulombic efficiency of 53.5% and an outstanding capacitance retention of 91%. Our research pointed out a promising direction to develop sustainable electrode materials for future high-performance supercapacitors.

## 1. Introduction

The ever-growing quest to meet global energy demand and to offset the challenge associated with the exploitation of fossil fuel has made it critical to develop sustainable energy solutions and energy storage systems. Among electrical energy storage devices, supercapacitor has shown its attractiveness owing to fast charge–discharge characteristics, high power density, and excellent cycle stability [1,2,3].

Electrode materials play a critical role in determining the performance of supercapacitors [4]. Carbon materials have been widely used for supercapacitor electrode due to their excellent electrical conductivity, high chemical stability, and low cost. Electrospinning is a straightforward and universal technique to produce submicron and nanometer scale fibers from various materials [5]. Electrospun carbon nanofibers (ECNF) have demonstrated their promises as high-performance electrode materials for supercapacitor development due to their features such as high porosity, large specific surface area, adequate structural integrity, and good electrochemical performance over numerous charge/discharge cycles [6,7,8,9].

In recent years, the development of ECNF aerogel (sponge) materials for electrode purposes has led to notable advances in the realm of supercapacitors [10,11,12]. Aerogel is an ultra-porous and network-structured solid material in which gaseous phase are predominant instead of liquid phase as in regular gel [13]. Aerogel possesses a broad spectrum of physical and chemical properties, making it highly promising for energy-related uses [14]. Electrospun nanofibers have brought tremendous promises in applications of aerogel materials since their first practice in 2014 due to the gelation-free synthesis of electrospun nanofibrous aerogels as well as their controllable morphology, structure, composition, and functionality [12]. The interconnected three-dimensional aerogel structure from ECNF can provide notable porosity and much increased ion-accessible specific surface area, which can consequently enhance the charge transfer between electrode and electrolyte in electrochemical operation and thus promote electrochemical performance of supercapacitors [15,16,17].

At present, sustainability has become an overarching driving force in research of electrochemical energy storage systems. Hence, the use of natural resources to develop economic and environmentally friendly carbon materials is favorable in supercapacitor electrode material design. For example, Cao et al. developed a nitrogen-doped carbon aerogel electrode material with a multi-channel structure at a micro/nano scale for supercapacitors from co-axial electrospinning of lignin with polymethyl methacrylate (PMMA) as a core solution and polyacrylonitrile (PAN) as shell solution followed by aerogel processing and subsequent carbonization [18]. The electrode material exhibited a maximum specific capacitance of 282 F/g at current density of 0.2 A/g in 6 M KOH electrolyte. Ding and collaborators demonstrated the use of activated biochar from millet straw to create an electrode material for supercapacitor with a specific surface area of 1264.8 m^2^/g and a pore volume of 0.954 cm^3^/g, resulting in a specific capacitance of 144 F/g at a current density of 0.2 A/g in 2 M KOH electrolyte [19]. Husain et al. prepared a biochar electrode material for supercapacitor from garden waste through pyrolysis [20]. The biochar produced at 800 °C showed a specific capacitance of 228 F/g at 1 A/g in 1 M H_2_SO_4_. Xu et al. further integrated a biochar from hydrothermal liquefaction of corn stover into ECNF aerogel structure and evaluated the resultant material as an electrode material for supercapacitor application [21]. This electrode material exhibited a specific capacitance up to 225 F/g at a current density of 0.5 A/g in 6 M KOH electrolyte.

In this research, our goal is to augment the electrochemical performance of biochar-integrated carbon nanofibrous aerogel material for supercapacitor application. Among all the biochar, wood biochar stands out as a highly promising material for supercapacitor electrode due to its remarkable attributes, like abundant pores and extensive surface area, which enable swift storage and release of electrical charges [22,23]. It is known that fluorine (F)-doping of carbon material forms semi-ionic C-F bonds, which can promote electric conductivity and lead to better electrochemical performance of the carbon material. The highly electronegative nature of F atoms can increase stability of the resultant carbon electrode for long-term supercapacitor performance [24,25]. In the meantime, carbon activation by using potassium hydroxide (KOH) can further increase the porous structure in carbon [26,27] and boost electrochemical performance of the resultant carbon electrode material for supercapacitor purpose. Herein we presented a novel approach to prepare a unique meso-microporous carbon electrode material for sustainable and high-performance supercapacitor development by combining the following processes: (1) F-treatment of wood biochar (a sustainable carbon resource); (2) integration of the F-treated wood biochar in electrospinning of PAN; (3) aerogel construction using the F-treated wood biochar-integrated electrospun PAN nanofibers; and (4) carbonization of the prepared aerogel material followed by carbon activation. This is the first-time demonstration that the combined processing as mentioned above can lead to a biochar-containing carbon nanofibrous aerogel electrode material with enormous increase in both micropore and mesopore volumes and consequently exceptional electrochemical capability, which outperformed the state-of-the-art electrochemical performance of biochar-containing carbon nanofibrous aerogel electrode materials.

## 2. Results and Discussion

### 2.1. Non-Activated Electrode Materials

#### 2.1.1. Morphology

**Figure 1:**
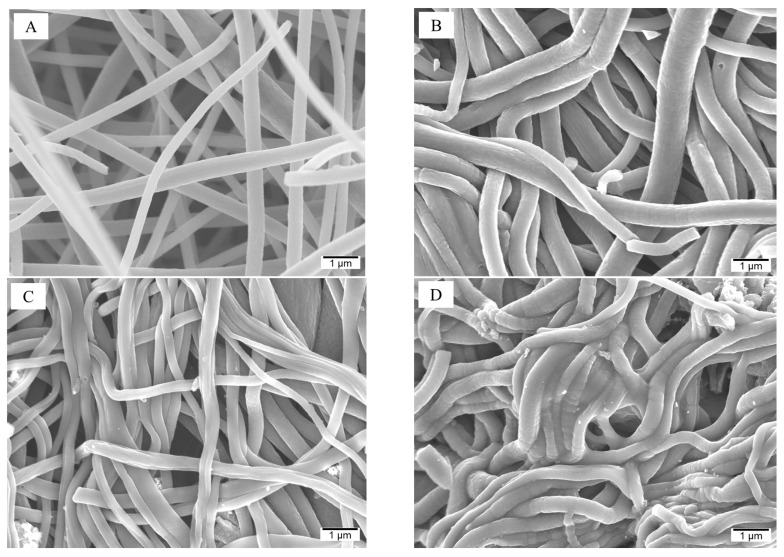

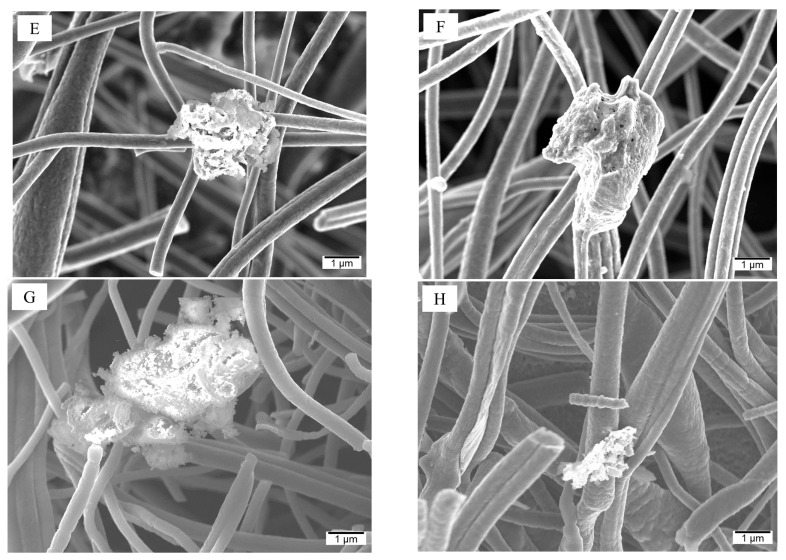
SEM images of carbon nanofiber and carbon nanofibrous aerogel materials: (**A**) ECNF; (**B**) ENFA; (**C**) B-ENFA; (**D**) FB-ENFA; (**E**) ECNF-B; (**F**) ECNF-FB; (**G**) ENFA-B; (**H**) ENFA-FB.

The carbon nanofibrous mat from electrospinning of PAN followed by stabilization and carbonization was composed of randomly deposited cylindrical shape fibers with relatively uniform size (~400 nm) (Figure 1A). The carbon nanofibers became densely packed in the constructed aerogel (ENFA) due to the freeze-drying process and crosslinking between nanofibers (Figure 1B–D). Including biochar and F-treated biochar in electrospun carbon nanofibers (ECNF) increased their surface roughness as shown in the ECNF-B and ECNF-FB nanofibers and resultant aerogels (Figure 1E–H). All the sample names are explained in the experimental part.

#### 2.1.2. Structure

**Figure 2:**
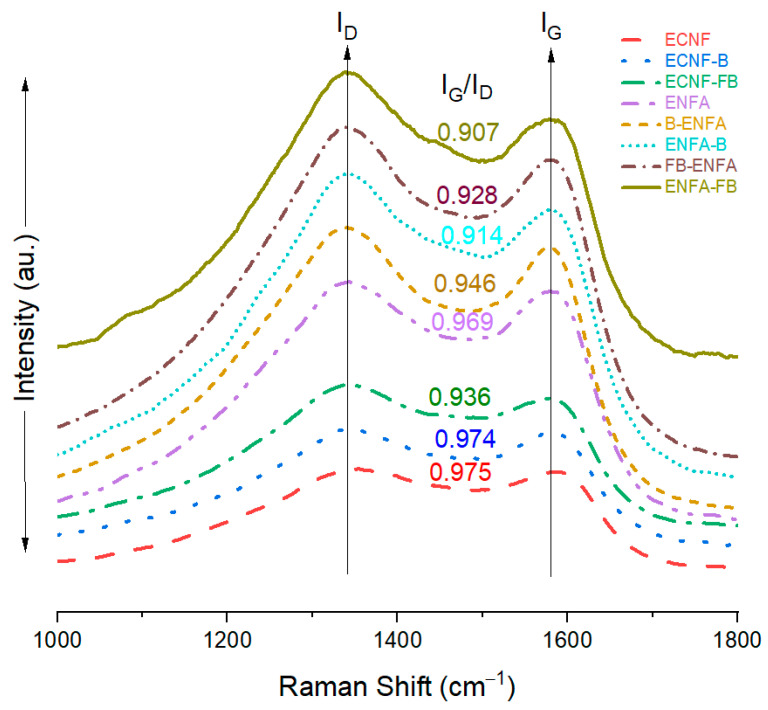
Raman spectra of carbon nanofiber and carbon nanofibrous aerogel materials.

Raman spectroscopy was used to examine the carbon structure of all the carbon nanofiber and aerogel materials (Figure 2). The Raman spectra of carbon materials generally show the D-band positioned around 1340 cm^−1^, which relates to the disordered construction of graphitized carbon structures, and the G-band positioned around 1580 cm^−1^, which is associated with organized graphitic form [28,29,30]. The positions of these two bands are independent of carbonization temperature, and the intensity ratio of G-band to D-band (I_G_/I_D_) generally indicates the proportion of structurally ordered graphite crystallites in overall carbon structure, and a high I_G_/I_D_ ratio indicates more sp^2^ hybridized ordered carbon structure in corresponding carbon material.

Compared to the I_G_/I_D_ ratio of ECNF (0.975), all the biochar-integrated nanofibers as well as aerogel materials showed lower I_G_/I_D_ ratios, indicating more disordered carbon structure upon the material processing including integration of biochar, F-treatment of biochar, and aerogel processing. In particular, the type II aerogel processing by using electrospun carbon nanofibers with embedded biochar (ENFA-B and ENFA-FB) showed even lower I_G_/I_D_ ratios in comparison with the type I aerogel processing by blending the biochar with electrospun carbon nanofibers (B-ENFA and FB-ENFA). ENFA-FB exhibited the lowest I_G_/I_D_ ratio (0.907), indicating the most disordered carbon structure among all the studied carbon materials.

**Figure 3:**
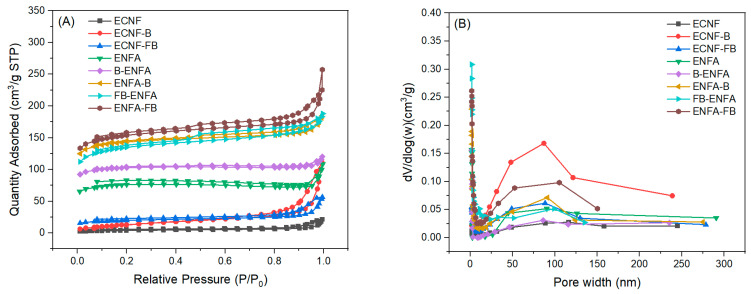
N_2_ adsorption/desorption isotherms (**A**) and pore size distribution (**B**) of carbon nanofiber and carbon nanofibrous aerogel materials.

The average pore width, pore volume, and BET surface area of all the carbon nanofibrous samples were determined using BET surface area analysis (Table 1 and Figure 3). Compared to ECNF, biochar integration in electrospun nanofibers, F-treatment of biochar, and aerogel processing could improve specific surface area, reduce pore size, and increase pore volume of the resultant carbon nanofibrous materials. It is noteworthy that aerogel processing significantly increased the micropore volume. Regarding the increase in specific surface area and pore volume, the type II aerogel processing outperformed the type I aerogel processing. In particular, the combination of type II aerogel processing with F-treated biochar (ENFA-FB) showed the largest BET surface area of ~472 m^2^/g with the maximum mesopore volume (0.11 cm^3^/g), macropore volume (0.035 cm^3^/g) and total pore volume (0.16 cm^3^/g).

#### 2.1.3. Electrochemical Performance

**Figure 4:**
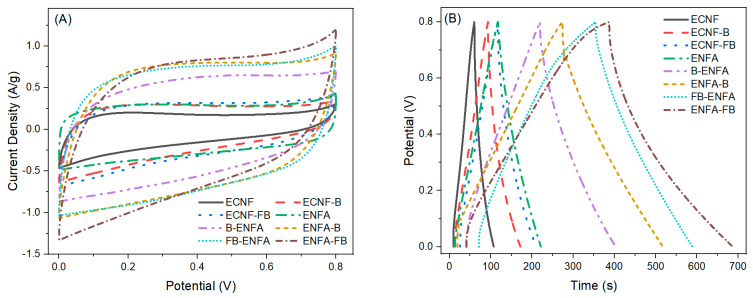

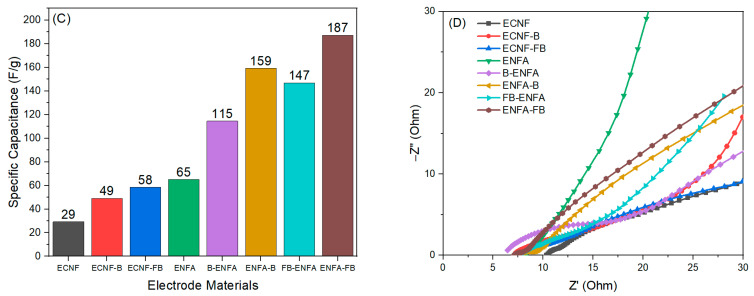
The CV curves at scan rate of 5 mV/s (**A**), GCD profiles at current density of 0.5 A/g (**B**), specific capacitances from GCD analysis at current density of 0.5 A/g (**C**), and Nyquist plots (**D**) of carbon nanofiber and carbon nanofibrous aerogel materials. The CV and GCD measurements were performed in 1 M H_2_SO_4_ with a potential window of 0–0.8 V.

The electrochemical performance of all the carbon nanofiber and carbon nanofibrous aerogel materials were evaluated by using cyclic voltammograms (CV) and galvanostatic charge-discharge (GCD) tests using a three-electrode setup (Figure 4).

The specific capacitance of neat carbon nanofiber (ECNF) was 29 F/g. Including biochar into ENCF (ECNF-B) increased its specific capacitance by 69% while the inclusion of F-treated biochar (ECNF-FB) doubled the ECNF’s specific capacitance. Compared to ECNF, the carbon nanofibrous aerogel structure (ENFA) could lead to more than doubled specific capacitance (65 F/g). Integration of biochar in the aerogel structure greatly increased the electrochemical performance of the resultant electrode materials and the use of F-treated biochar could further increase the electrochemical performance of corresponding electrode materials. Compared to ENFA, the specific capacitance of B-ENFA and FB-ENFA increased by 77% and 126%, respectively. It is noteworthy that type II aerogel processing, i.e., using biochar-embedded ECNF, outperformed the type I aerogel processing, i.e., blending biochar with ECNF, in electrochemical performance. Compared to B-ENFA and FB-ENFA (type I), the specific capacitance of ENFA-B and ENFA-FB (type II) increased by 38% and 27%, respectively. The largest specific capacitance was observed with ENFA-FB and reached 187 F/g, more than six times that of ECNF.

Electrochemical impedance spectroscopy (EIS) was used to further analyze the electrode materials (Figure 4D). Compared to ECNF, the biochar-integrated carbon nanofibrous aerogel materials exhibited enhanced capacitive behavior, as evidenced by a wider angle between the straight line of respective Nyquist plot and the axis of the real part of complex impedance. In the meantime, the biochar-integrated aerogel materials had lower electrode resistance, as evidenced by the smaller intercept at the axis of the real part of complex impedance in respective Nyquist plot. The observation from EIS is consistent with the results of specific capacitance.

The enhancement of electrochemical performance of carbon nanofibrous electrode materials by integrating with biochar, F-treatment of biochar, and aerogel processing can be firstly attributed to the increased fiber surface roughness, specific surface area, and pore volume, making it easier for electrolyte ions to penetrate into the electrode [19]. Secondly, the material processing as mentioned above could increase the disordered carbon structure and thus add the number of active sites for charge transfer, benefiting the electrochemical performance [31]. Thirdly, F-treatment could promote repulsive interaction inside the carbon lattice and result in a larger number of active sites and simultaneous stabilization of carbon lattice’s edge position, boosting the specific capacitance [32]. Additionally, the formation of semi-ionic C-F bonds could enhance electrical conductivity and electrolyte wettability of the electrode materials.

To explore the maximum potential of the best electrode material so far, i.e., ENFA-FB, we extended the potential window to 0–1 V. It is found that ENFA-FB achieved an even better specific capacitance of 215 F/g at current density of 0.5 A/g through GCD analysis with this potential window. Therefore, in the following part we used the potential window of 0–1 V to characterize the electrochemical performance of activated aerogel electrode materials.

### 2.2. Electrode Materials from Activated Aerogels

Chemical activation is a well-known efficient way to increase specific surface area of carbon materials [33]. To further explore the maximum potential of the biochar-integrated carbon nanofibrous aerogel electrode materials for supercapacitor application, we performed carbon activation to ENFA, ENFA-B, and ENFA-FB materials using KOH, characterized the activated aerogels (ENFAa, ENFA-Ba, and ENFA-FBa), and evaluated their electrochemical performance.

#### 2.2.1. Morphology

**Figure 5:**
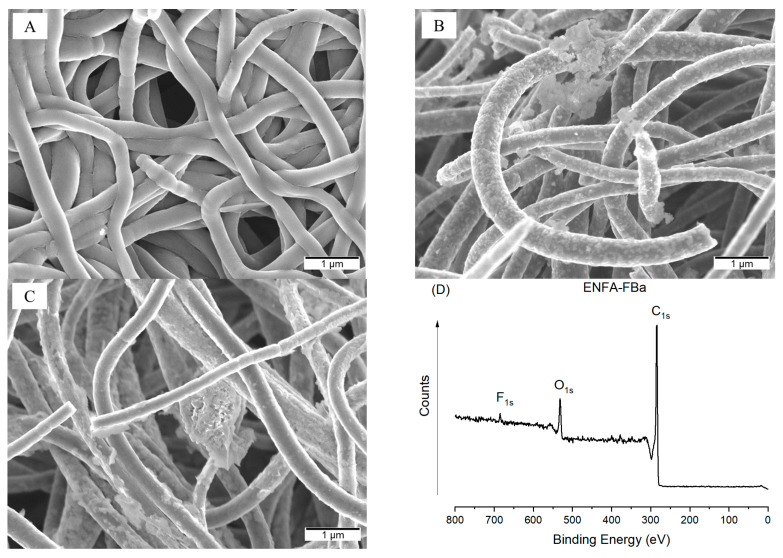
SEM images of activated carbon nanofibrous aerogels: (**A**) ENFAa; (**B**) ENFA-Ba; (**C**) ENFA-FBa; and XPS spectrum of ENFA-FBa (**D**).

After activation, the carbon nanofibrous aerogels maintained fibrous and aerogel structure but slightly smaller average fiber size (~350 nm) (Figure 5A–C). The biochar-integrated carbon nanofibrous aerogels (ENFA-Ba and ENFA-FBa) demonstrated much more roughness on fiber surface than their counterparts before activation.

#### 2.2.2. Structure

Upon activation with KOH, the I_G_/I_D_ of all the carbon nanofibrous aerogels reduced, e.g., the I_G_/I_D_ of ENFA-FB reduced from 0.907 to 0.898 after activation, indicating that the activation increased the disordered carbon structure. In the meantime, the XPS result confirmed the presence of F element, but in a small amount, e.g., atomic percentage of F is ~1.0 at.% in the case of ENFA-FBa (Figure 5D).

**Figure 6:**
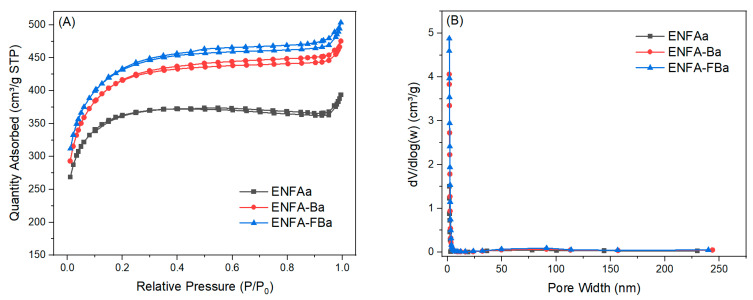
N_2_ adsorption/desorption isotherms (**A**) and pore size distribution (**B**) of activated carbon nanofibrous aerogel materials.

After activation, there is a significant increase in specific surface area and pore volume, particularly micropore volume and mesopore volume, of the carbon nanofibrous aerogels (Table 2 and Figure 6 vs. Table 1 and Figure 3). The integration of biochar in the carbon nanofibrous aerogel (ENFA) resulted in an even more significant increase in micropore and mesopore volumes upon activation. Among all the studied carbon nanofibrous materials in this research, the activated carbon nanofibrous aerogel with F-treated biochar (ENFA-FBa) possessed the largest specific surface area (1375 m^2^/g), the smallest average pore size (2.3 nm), and the largest pore volume (0.73 cm^3^/g), i.e., an increase of ~190%, a reduction of ~60%, and an increase of ~350%, respectively, when compared to its counterpart before activation (ENFA-FB). Remarkably, the micropore volume and mesopore volume of ENFA-FB increased by more than 18 times and more than 240%, respectively, upon activation (ENFA-FBa), indicating a distinctive meso-microporous feature.

The carbon activation with KOH is based on carbon etching [34]. Apparently, the biochar, in particular, the F-treated biochar, greatly enhanced the pore formation in the activation process. The inclusion of biochar in electrospun nanofibers could create voids during the volume-shrinking carbonization process of PAN nanofibers, and thus increase the pore volume of resultant carbon nanofibers, which in turn facilitated carbon etching in the activation process.

#### 2.2.3. Electrochemical Performance

##### Three-Electrode Analysis

**Figure 7:**
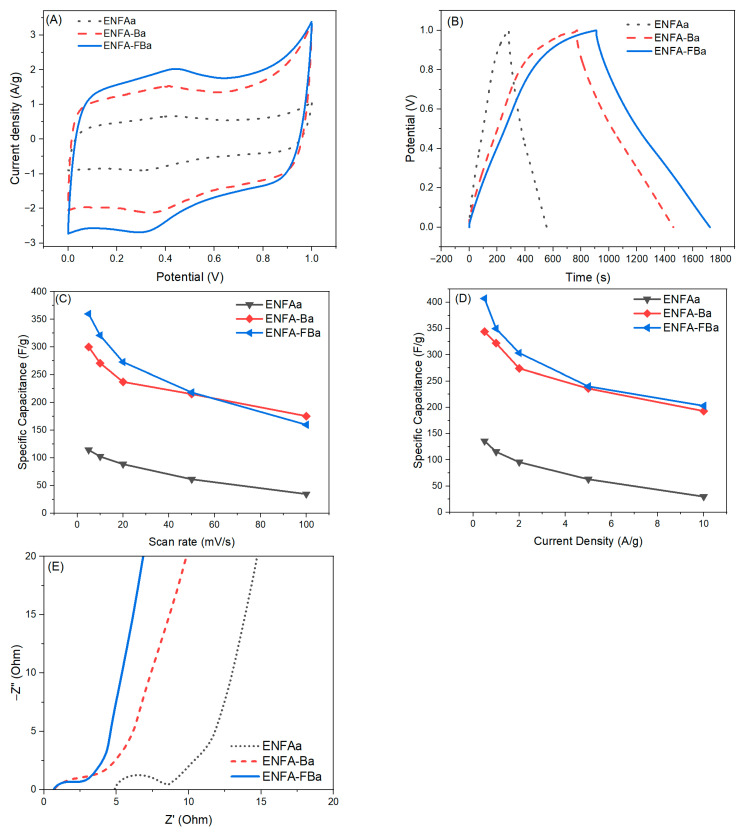
The CV curves at scan rate of 5 mV/s (**A**), GCD profiles at current density of 0.5 A/g (**B**), specific capacitances from CV at various scan rates (**C**), specific capacitances from GCD at various current densities (**D**), and Nyquist plots (**E**) of the activated carbon nanofibrous aerogels.

The electrochemical performance of the activated carbon nanofibrous aerogels, i.e., ENFAa, ENFA-Ba, and ENFA-FBa, were first evaluated by using CV and GCD tests through a three-electrode setup (Figure 7A,B). All these activated carbon nanofibrous aerogel electrode materials had CV curves with quasi-rectangular shapes, a sign of good electric double layer capacitive behavior. Compared to the non-activated counterparts, all activated carbon nanofibrous aerogels showed much improved electrochemical performance. From both the CV and GCD tests, ENFA-FBa exhibited the largest specific capacitance among all the tested electrode materials in this research, i.e., 360 F/g at scan rate of 5 mV/s and 407 F/g at current density of 0.5 A/g, which is ~20% more than that of ENFA-Ba, three times that of ENFAa, and two times that of the non-activated counterpart (ENFA-FB) (Figure 7C,D). To further understand the activated electrode materials, we performed EIS analysis on ENFAa, ENFA-Ba, and ENFA-FBa (Figure 7E). According to the Nyquist plots, ENFA-FBa showed the smallest electrolyte resistance (the smallest semi-circle in high frequency range), the least electrode resistance (the smallest intercept at the axis of the real part of complex impedance), and the best capacitive behavior (the largest angle between the straight line of the Nyquist profile and the axis of the real part of complex impedance).

It is noteworthy that the largest specific capacitance of ENFA-FBa reached 407 F/g. This is significantly higher than the maximum specific capacitance of the electrospun carbon nanofibrous aerogel materials as reported in past years (<300 F/g upon three-electrode tests) [16,18,35], indicating the significance of the combination of biochar integration in electrospun carbon nanofibers, F-treatment of biochar, 3D aerogel construction, and carbon activation in improving electrochemical performance of the carbon nanofibrous electrode materials for supercapacitor uses. The superior electrochemical performance of ENFA-FBa could be attributed to the much-improved micropore volume, mesopore volume, and specific surface area as well as more disordered carbon structure upon activation in the presence of F-treated biochar, which contributed much more active sites to charge storage and significantly increased the electrochemical interaction with electrolyte.

##### Two-Electrode Analysis

**Figure 8:**
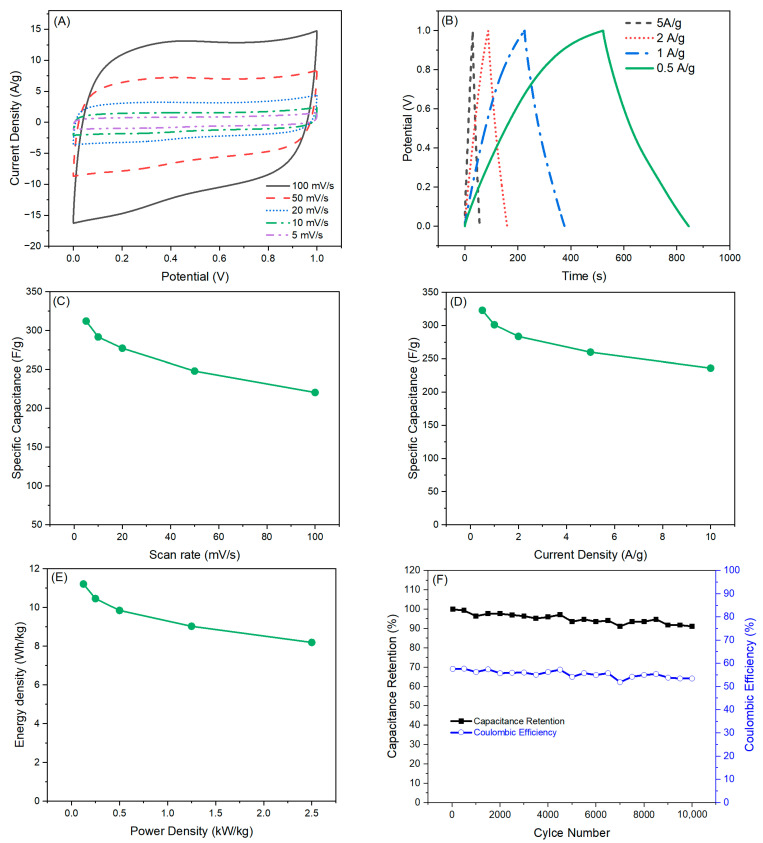
Electrochemical evaluation of the activated carbon nanofibrous aerogel electrode material with embedded F-doped biochar in nanofibers (ENFA-FBa) in a symmetric two-electrode cell: (**A**) CV curves at various scan rates; (**B**) GCD profiles at various current densities; (**C**) specific capacitances from CV at various scan rates; (**D**) specific capacitances from GCD at various current densities; (**E**) energy density vs. power density; (**F**) cycle stability and coulomb efficiency up to 10,000 cycles of charging-discharging at current density of 10 A/g.

To evaluate the practical use of the best carbon nanofibrous aerogel material (ENFA-FBa), a symmetric two-electrode cell was assembled and tested (Figure 8).

The quasi-rectangular CV curves of the device, particularly at low scan rates, indicated good capacitive performance (Figure 8A). The charge/discharge profile of the device showed isosceles shape at high current densities but a non-linear charging profile at low current densities especially at 0.5 A/g (Figure 8B). This is probably due to the meso-microporous structure of ENFA-FBa, having simultaneously the largest micropore volume, mesopore volume, and total pore volume among all the studied carbon nanofibrous electrode materials. At low current density, it took more time for electrolyte ions to transport and access all the available surface of the electrode material. The formation of electrical double layer of electrolyte ions on the surface of meso- and macropores was relatively easy but the electrolyte ions had difficulty to transport into micropores and access their surface for charge storage, which led to deviation from the linearity of charging. The meso-microporous electrode material is ideal for the supercapacitor designed for the accumulation of maximum capacity and operation in relatively long-time range [36].

**Figure 9:**
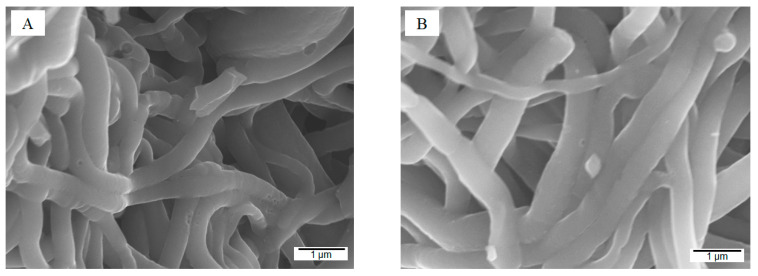
SEM images of the ENFA-FBa electrode material before (**A**) and after (**B**) cycle test.

The manufactured ENFA-FBa device showed superior specific capacitances of 312, 292, 277, 248, and 220 F/g at scan rates of 5, 10, 20, 50, and 100 mV/s, respectively, from CV analysis (Figure 8C). The device showed even better specific capacitances of 323, 301, 284, 260, and 236 F/g, at current densities of 0.5, 1, 2, 5, and 10 A/g, respectively, from GCD analysis (Figure 8D). At a very high current density of 50 A/g, the device still retained 40% of its specific capacitance at current density of 0.5 A/g, i.e., 130 F/g, indicating excellent rate stability. The device’s maximum energy density reached 11.2 Wh/kg at 125 W/kg power density (Figure 8E). After 10,000 cycles of charging-discharging at current density of 10 A/g, the ENFA-FBa device demonstrated a capacitance retention of 91% (Figure 8F), indicating exceptional cycle stability even at high current density. In addition, the device showed a stable coulombic efficiency (53.5%), suggesting a stable long-term performance. The SEM images of the ENFA-FBa electrode material before and after cycle test (Figure 9) did not show appreciable morphological change, another indication of its stability in practice. The presence of F element in ENFA-FBa may have contributed to this excellent cycle stability.

## 3. Conclusions

This research demonstrated a facile and powerful approach to prepare superior carbon nanofibrous supercapacitor electrode materials through a series of sequential processing including F-treatment of wood biochar, incorporation of the biochar in electrospinning of polyacrylonitrile (PAN), aerogel construction with the F-treated biochar-embedded electrospun PAN nanofibers, and carbonization followed by carbon activation.

The activated carbon nanofibrous aerogel with F-treated biochar in carbon nanofibers (ENFA-FBa) demonstrated outstanding electrochemical performance as supercapacitor electrode material. ENFA-FBa showed the highest specific capacitance among all the reported biochar-containing electrospun carbon nanofibrous aerogel electrode materials for supercapacitor uses up to date, i.e., 407 F/g at 0.5 A/g current density, through three-electrode electrochemical characterization in 1 M H_2_SO_4_ electrolyte. A two-electrode device (cell) with ENFA-FBa as electrode material also demonstrated exceptional electrochemical performance in terms of practical evaluation with a specific capacitance of 323 F/g at 0.5 A/g current density and an energy density of 11.2 Wh/kg at 125 W/kg of power density. The ENFA-FBa device further exhibited a consistent coulombic efficiency of 53.5% and an outstanding capacitance retention of 91% after 10,000 cycles of charging-discharging at 10 A/g.

The extraordinary capacitive performance of ENFA-FBa electrode material could be mainly attributed to the unique meso-microporous structure of ENFA-FBa, a result of the sequential processes as indicated above. ENFA-FBa possessed simultaneously the largest micropore volume, mesopore volume, and total pore volume among all the studied carbon nanofibrous electrode materials in this research. Together with more disordered carbon structure upon activation in the presence of F-treated biochar, the ENFA-FBa electrode material acquired much more active sites to charge storage and significantly increased the electrochemical interaction with electrolyte, as evidenced by the smallest electrolyte resistance and the least electrode resistance from EIS test.

Overall, our research provided valuable insights into the development of sustainable carbon electrode materials for future supercapacitor applications from the point of view of long-term performance, cost-effectiveness, and environmental benefit.

## 4. Materials and Methods

### 4.1. Materials

Polyacrylonitrile (PAN, average molecular weight Mw = 150,000), N, N-Dimethylformamide (DMF), ethyl alcohol, tert-butanol (anhydrous), potassium hydroxide (KOH), 1,4 Benzoxazin-3-one (BO), polyvinyl alcohol (PVA), hydrofluoric acid (HF), hydrochloric acid (HCl), and sulfuric acid (H_2_SO_4_) at reagent grade were purchased from Sigma–Aldrich (St. Louis, MO, USA). Oakwood biomass was employed as the starting material for biochar preparation. Stainless steel mesh (316 grade) was used as current collector for electrochemical evaluation.

### 4.2. Biochar Processing

The oakwood feedstock was first dried at 50 °C to remove moisture and loaded into a tubular reactor. The wood was next heated to 500 °C with a heating rate of 10 °C/min in a N_2_ atmosphere with a flow rate of 400 mL/min, held at 500 °C for 30 min, and then cooled down to room temperature to obtain the biochar. Before use, the biochar was activated at 800 °C for 1 h in a nitrogen environment with KOH in a mass ratio of 1:1. The activated biochar was washed with HCl and dried at 120 °C. To make F-treated biochar, the raw wood feedstock was pretreated with a 20 wt.% concentrated hydrofluoric acid followed by centrifuging, washing with DI water, and drying at 120 °C. Afterwards the wood feedstock went through the same biochar processing as described above.

### 4.3. Electrospinning and Carbonization

A 10 wt.% PAN solution in DMF with 1 wt.% BO was prepared and loaded in a 10 mL syringe with an 18-gauge stainless steel needle for electrospinning. The electrospinning was carried out with a voltage of 15 kV and solution loading rate of 1 mL/h. The distance between the needle tip and the grounded aluminum foil-wrapped collector was maintained at 15 cm. The as-collected nanofibrous mat was removed from the aluminum foil and dried in air for 24 h at ambient temperature. To fabricate the nanofibers with biochar, a 10 wt.% PAN-DMF solution with 1 wt.% BO and 2 wt.% biochar (pristine or F-treated) was used for electrospinning.

To create carbon nanofibers, these PAN-based nanofibers were stabilized in air at 250 °C for 6 h and then carbonized at 900 °C for 1 h in N_2_ atmosphere. The carbon nanofiber from the electrospun PAN nanofiber was denoted as ECNF while the carbon nanofiber from the electrospun PAN nanofiber with biochar and F-treated biochar was denoted as ECNF-B and ECNF-FB, respectively.

### 4.4. Preparation of Carbon Nanofibrous Aerogels

To make carbon nanofibrous aerogel, PAN nanofibrous aerogel was prepared first. The obtained electrospun PAN nanofibrous mat was cut into small pieces (1 cm × 1 cm) and homogenized in water with some ethanol/tert-butanol by using a homogenizer (T25, IKA Works, Wilmington, NC, USA). The dispersion was centrifuged and washed with fresh DI water three times to remove organic solvents. Then ~30 mL of the homogenized aqueous dispersion was kept in a freezer overnight and finally freeze-dried for 48 h.

To make PAN nanofibrous aerogels with biochar, two aerogel processing methods were applied. In type-I aerogel processing, a blend of electrospun PAN nanofibrous mat and biochar at 5:1 mass ratio (PAN nanofiber: biochar) was homogenized in the medium of water and ethanol/tert-butanol and subsequently freeze-dried as described above. In type-II aerogel processing, the electrospun PAN nanofibrous mat with biochar inside by integrating biochar in PAN electrospinning solution was used following the abovementioned homogenization and freeze-drying process.

The final carbon nanofibrous aerogels were obtained by carbonizing corresponding PAN nanofibrous aerogels following the same stabilization and carbonization procedures as described earlier for PAN nanofiber carbonization. The carbon nanofibrous aerogel without biochar was denoted as ENFA. The carbon nanofibrous aerogels with biochar and F-treated biochar from type-I aerogel processing were denoted as B-ENFA and FB-ENFA, respectively. The carbon nanofibrous aerogels with biochar and F-treated biochar from type-II aerogel processing were denoted as ENFA-B and ENFA-FB, respectively.

### 4.5. Activation of the Carbon Nanofibrous Aerogels

The carbon nanofibrous aerogel materials (ENFA, ENFA-B, and ENFA-FB) were individually combined with KOH activation agent at a mass ratio of 1:1 and activated at 800 °C for 1 h in a nitrogen environment. Upon cooling down to room temperature, the activated aerogels were thoroughly cleaned with 3 M HCl and DI water and dried at 120 °C. These activated carbon nanofibrous aerogels were denoted as ENFAa, ENFA-Ba, ENFA-FBa, respectively.

### 4.6. Characterization

A scanning electron microscope Auriga-BU FIB FESEM (Carl Zeiss, White Plains, NY, USA) was used to study the morphology of the prepared carbon nanofiber (ECNF) and aerogel (ENFA) materials. After being degassed at 300 °C for 6 h, the specific surface area and pore volume of the ECNF and ENFA samples were examined by nitrogen adsorption using an ASAP 2020 surface area and porosity analyzer (Micromeritics, Norcross, GA, USA). The carbon structure was studied using an XploRA Raman confocal microscope (Horiba, Piscataway, NJ, USA) with a wavelength of 532 nm. Surface elemental composition of the carbon nanofibrous aerogel material was examined via an X-ray photon spectrometer (XPS, Thermo Scientific Escalab Xi+ (Waltham, MA, USA)).

A 660E workstation (CH Instruments, Austin, TX, USA) was used to investigate the electrochemical performance of all the electrode materials in 1 M H_2_SO_4_ electrolyte solution with potential window of 0–0.8 V or 0–1 V using a three-electrode setup that included an Au working electrode attaching to the electrode material (1 cm × 1 cm), a Pt wire counter electrode, and an Ag/AgCl reference electrode. For aerogel materials, specifically, the working electrode material with a thickness of ~25 µm was prepared by coating a stainless-steel mesh (316 grade) with a slurry made with 80 wt.% respective aerogel material (~0.8 mg), 10 wt.% acetylene black, 10 wt.% polyvinylidene fluoride (PVDF), and a few drops of N-methyl-2-pyrrolidone (NMP) solvent. Cyclic voltammograms (CV), galvanostatic charge-discharge (GCD), and electrochemical impedance spectroscopy (EIS) were performed. The specific capacitance of electrode materials (C) was calculated from CV curves at 5 mV/s using Equation (1).

(1) $$C=\frac{1}{2mv(V_{t}-V_{0})}\;\int_{V_{0}}^{V_{t}}I(V)dV$$

where *m* is mass of electrode material (g), *I*(*V*) is current (A), *v* is scan rate (mV/s), and *V*_0_ and *V_t_* are the lower and upper potential limits of the chosen potential window, respectively.

Alternatively, the specific capacitance of the electrode materials was calculated using the discharge portion of the GCD curves using Equation (2).

(2) $$C=\frac{I∆t}{m∆V}$$

where *I* is discharge current (A), Δ*t* is discharge time (s), Δ*V* is potential window (V), and *m* is the mass of electrode material (g).

A symmetric two-electrode setup (a cell) was further used to evaluate the device performance of the prepared electrode material. Two pieces of equal mass electrode material (1 cm × 1 cm) were loaded in an MTI split cell (EQ-STC) with Whatman filter paper as separator and 1 M H_2_SO_4_ as electrolyte. Electrochemical evaluation was conducted by using the CHI 660E workstation. In the case of CV test, the specific capacitance of the electrode material was calculated by multiplying two from Equation (1) using the mass of single electrode material (Equation (3)).

(3) $$C=\frac{1}{mv(V_{t}-V_{0})}\;\int_{V_{0}}^{V_{t}}I\left(V\right)dV\;$$

where *I*(*V*) is current (A), *v* is scan rate (mV/s), and *V*_0_ and *V_t_* are the lower and upper potential limits of the chosen potential window (V), and *m* is mass of single electrode material.

The specific capacitance of electrode material from GCD test was calculated using Equation (4).

(4) $$C=\frac{2I∆t}{m\Delta V}$$

where *I* is discharge current (A), Δ*t* is discharge time (s), *m* is mass of single electrode material (g), and Δ*V* is potential window.

The energy density of the prepared device (cell) (*E*, Wh/kg) was calculated using Equation (5).

(5) $$E=\frac{1}{8\times 3.6}\times C\times ({\Delta V)}^{2}$$

where *C* is the specific capacitance of the electrode material from the GCD test and Δ*V* is potential window.

The power density *P* (W/kg) of the device was calculated from Equation (6).

(6) $$P=\frac{E\times 3600}{t}$$

where *E* is energy density of the cell (Wh/kg) and *t* is discharge time (s).

## Figures and Tables

**Table 1:** BET surface area and porosity of carbon nanofiber and carbon nanofibrous aerogel materials.

Sample Name	BET Surface Area	Average Pore Size	V_mic_	V_mes_	V_mac_	V_t_	%V_mic_	%V_mes_	%V_mac_
	m^2^/g	nm	cm^3^/g	cm^3^/g	cm^3^/g	cm^3^/g	%	%	%
ECNF	13.8	14.9	0.0011	0.0135	0.0149	0.02951	3.8	45.82	50.38
ECNF-B	50.4	15.5	0.003	0.0927	0.0684	0.1641	1.83	56.49	41.67
ECNF-FB	63.1	10.1	0.0035	0.0408	0.0253	0.06963	5.01	58.61	36.38
ENFA	229	7.8	0.01	0.0225	0.0346	0.06722	14.97	33.50	51.53
B-ENFA	309	5.0	0.0089	0.0175	0.0168	0.04322	20.63	40.55	38.82
ENFA-B	435	5.1	0.0132	0.0812	0.0067	0.1012	13.07	80.33	6.60
FB-ENFA	417	4.4	0.0185	0.0935	0.0163	0.1284	14.44	72.85	12.71
ENFA-FB	472	5.8	0.0167	0.1085	0.0352	0.1605	10.38	67.66	21.95

V_mic_: micropore volume; V_mes_: mesopore volume; V_mac_: macropore volume; V_t_: total pore volume; %V_mic_: percentage of micropore volume in total volume; %V_mes_: percentage of mesopore volume in total volume; %V_mac_: percentage of macropore volume in total volume.

**Table 2:** BET surface area and porosity of activated carbon nanofibrous aerogels.

Sample Name	BET Surface Area	Average Pore Size	V_mic_	V_mes_	V_mac_	V_t_	%V_mic_	%V_mes_	%V_mac_
	m^2^/g	nm	cm^3^/g	cm^3^/g	cm^3^/g	cm^3^/g	%	%	%
ENFAa	1137	2.5	0.1155	0.0521	0.0243	0.1919	60.18	27.13	12.69
ENFA-Ba	1319	2.3	0.3113	0.2513	0.0331	0.5957	52.25	42.19	5.56
ENFA-FBa	1375	2.3	0.3262	0.3719	0.0367	0.7348	44.39	50.61	5.0

V_mic_: micropore volume; V_mes_: mesopore volume; V_mac_: macropore volume; V_t_: total pore volume; %V_mic_: percentage of micropore volume in total volume; %V_mes_: percentage of mesopore volume in total volume; %V_mac_: percentage of macropore volume in total volume.

## Data Availability

The data presented in this study are openly available in article.
